# Prognosis of Asymptomatic Intracranial Stenosis in Patients With Transient Ischemic Attack and Minor Stroke

**DOI:** 10.1001/jamaneurol.2020.1326

**Published:** 2020-05-26

**Authors:** Robert Hurford, Frank J. Wolters, Linxin Li, Kui Kai Lau, Wilhelm Küker, Peter M. Rothwell

**Affiliations:** 1 Wolfson Centre for the Prevention of Stroke and Dementia, Nuffield Department of Clinical Neurosciences, University of Oxford, Oxford, England

## Abstract

**Importance:**

Asymptomatic intracranial stenosis (ICS) is a frequent finding on imaging results, particularly in the assessment of acute stroke. Although the management of symptomatic ICS is informed by randomized trials, to our knowledge there are few data on the prevalence and prognosis of asymptomatic ICS in patients with stroke and transient ischemic attack (TIA).

**Objective:**

To study the age-specific prevalence and prognosis of asymptomatic ICS in a population-based cohort of patients with TIA and minor stroke.

**Design, Setting, and Participants:**

All patients (predominantly white) recruited to the Oxford Vascular Study (Oxfordshire, England) between March 1, 2011, and March 1, 2018, with TIA and minor ischemic stroke (National Institutes of Health Stroke Scale score, ≤3), irrespective of age, were included (n = 1579). We determined the age-specific prevalence of 50% or more asymptomatic ICS and the associated stroke risk by face-to-face follow-up to 2018 on standard medical treatment without stenting.

**Exposures:**

Patients underwent magnetic resonance angiography of the intracranial and cervicocranial arteries, computed tomography angiography if magnetic resonance angiography was contraindicated, or carotid/transcranial Doppler ultrasonography if computed tomography angiography was contraindicated.

**Main Outcomes and Measures:**

The primary outcomes were the prevalence and prognosis of asymptomatic ICS.

**Results:**

Of 1368 eligible patients (mean [SD] age, 69.2 [13.9] years; 700 men [51.2%]) with intracranial vascular imaging, 426 ICS were identified in 260 patients (19.0%): 58 (4.2%) with only symptomatic ICS, 155 (11.3%) with only asymptomatic ICS, and 47 (3.4%) with both. The prevalence of any asymptomatic ICS increased from 4.8% for patients younger than 70 years to 34.6% for patients 90 years or older (*P* for trend < .001; odds ratio per decade, 1.96; 95% CI, 1.69-2.27) and was greater than that of 50% or more asymptomatic carotid bifurcation stenosis (202 [14.8%] vs 105 patients [7.2%]; relative risk, 2.04; 95% CI, 1.63-2.55, *P* < .001). However, the 155 patients with only asymptomatic ICS had no increase in risk of ischemic stroke compared with those with no ICS (unadjusted HR, 1.03, 95% CI, 0.49-2.17), with 8 first recurrent events (5.2%) during 506 patient-years of follow-up and 3 in the territory of the ICS (annualized risk, 0.59%; 95% CI, 0.12-1.73).

**Conclusions and Relevance:**

The prevalence of asymptomatic ICS increases with age in white patients with TIA and minor stroke and is greater than that of asymptomatic carotid stenosis, but asymptomatic ICS does not increase the short- or medium-term risk of distal recurrent ischemic stroke for patients receiving standard medical treatment.

## Key Points

**Question:**

What is the age-specific prevalence and prognosis of asymptomatic intracranial stenosis (ICS) in a population-based cohort of patients with transient ischemic attack (TIA) and minor stroke?

**Findings:**

In this cohort study of 1368 patients with TIA and minor stroke, asymptomatic ICS was identified in 202 patients (14.8%) overall, with higher prevalence at older ages, reaching 34.6% at 90 years or older. In 155 patients with only asymptomatic ICS (11.3%), there was no increase in risk of ischemic stroke compared with those with no ICS, with 8 first recurrent events (5.2%) during 506 patient-years of follow-up and an annualized risk of same-territory ischemic stroke of 0.59%.

**Meaning:**

The prevalence of asymptomatic ICS increases with age in patients with TIA and minor stroke and is greater than that of asymptomatic carotid stenosis, but asymptomatic ICS does not increase the short- or medium-term risk of distal recurrent ischemic stroke in patients receiving standard medical treatment.

## Teaser

This cohort study examines the age-specific prevalence and prognosis of asymptomatic intracranial stenosis in British patients with transient ischemic attack and minor stroke.

## Introduction

Intracranial stenosis (ICS) of the major cerebral arteries is an important cause of ischemic stroke.^1,2^ The management of recently symptomatic ICS has been established by randomized trials.^3,4^ However, although asymptomatic ICS is frequently encountered in clinical practice, particularly with the now widespread availability of magnetic resonance angiography (MRA) and computed tomography angiography (CTA), and its use in patients assessed for mechanical thrombectomy, to our knowledge there are few population-based data on the prevalence and prognosis in white patients with ischemic stroke/transient ischemic attack (TIA).^5,6^ Moreover, the prevalence of asymptomatic ICS reported in the literature is highly heterogeneous, likely because of the differing first-line imaging techniques between studies (including transcranial Doppler, MRA, and CTA) and definitions (eg, flow velocity criteria or various percentage reductions in luminal diameter).^7,8,9,10^ There are also relatively few studies conducted in white populations. One large study of 1765 community-dwelling individuals estimated the US prevalence of 50% or greater asymptomatic ICS for white people aged 65 to 90 years as 8%.^11^ In patients with stroke and TIA, the prevalence is reported to be higher; a hospital-based study of 403 patients with stroke admitted to a single French center reported an asymptomatic ICS rate of 18.4%.^10^

Similarly, there are relatively few data on the prognosis of asymptomatic ICS.^12,13,14^ One Spanish community-based cohort of 80 stroke-free participants, but with a high burden of vascular risk factors, reported a rate of 2.9% and 12.6% of ischemic stroke and any vascular event/vascular death, respectively, during a 7-year follow-up.^15^ In patients with stroke/TIA, a subset of participants recruited to the US-based Warfarin-Aspirin Symptomatic Intracranial Disease (WASID)^16^ trial with coexistent asymptomatic ICS had a 3.5% 1-year risk of same-territory ischemic stroke.^7^

We therefore performed what, to our knowledge, is the first population-based study of 50% or greater asymptomatic ICS in patients with stroke/TIA, irrespective of age, followed up while receiving intensive medical treatment. We aimed to determine the age-specific prevalence, predictors, and prognosis of asymptomatic ICS to enable clinicians to reliably counsel patients about these commonly encountered vascular abnormalities.

## Methods

We studied consecutive patients with TIA or minor ischemic stroke enrolled in the Oxford Vascular Study (OXVASC) between March 1, 2011, and March 1, 2018, with follow-up to September 28, 2018. OXVASC is a longitudinal population-based incidence cohort of all acute vascular events in a defined population of 92 728 covered by approximately 100 primary care physicians in 9 primary care practices in Oxfordshire, England. An estimated 97% of the true study residential population is registered with a primary care practice; most unregistered people are young students. The study area contains a mix of urban and rural populations. The OXVASC population is 94% white, 3% Asian, 2% Chinese, and 1% Afro-Caribbean.^17^

Detailed methods of OXVASC have been reported previously.^18^ Multiple overlapping methods were used for ascertaining all individuals with TIA and stroke, approaching 100% of events reaching medical attention. These include: (1) a daily, rapid-access clinic where participating general practitioners and the local emergency department refer individuals with suspected TIA or minor stroke; (2) daily searches of admissions to the medical, stroke, neurology, and other relevant wards; (3) daily searches of the local emergency department attendance register; (4) daily searches of in-hospital death records via the bereavement office; (5) monthly searches of all death certificates and coroner's reports for out-of-hospital deaths; (6) monthly searches of general practitioner diagnostic coding and hospital discharge codes; and (7) monthly searches of all brain and vascular imaging referrals.^19,20^ Patients gave written informed consent after an event or assent was obtained from a relative for patients who were unable to provide consent.

For this study, written informed consent or assent from relatives was obtained in all participants for study interview and follow-up, including ongoing review of primary care and hospital records and death certificate data. OXVASC was approved by the Oxfordshire research ethics committee.

All patients with TIA or minor ischemic stroke were eligible, irrespective of age or frailty. To maximize inclusion, we used multiple imaging modalities, with MRA as first choice, CTA if MRA was contraindicated (eg, implantable devices or claustrophobia), and transcranial Doppler and carotid ultrasonography if CTA was also contraindicated (eg, low glomerular filtration rate).

Demographic data and stroke risk factors were collected from face-to-face interviews by study physicians as soon as possible after referral or hospital admission and cross-referenced with primary care records. A detailed clinical history was recorded for all patients and assessments were made for stroke severity using the National Institute of Health Stroke Scale (NIHSS) as recorded on assessment. Minor stroke was defined as an NIHSS score of 3 or less. The cause of ischemic events was classified according to the Trial of Org 10172 in Acute Stroke Treatment criteria.^21^ Stroke and TIA were defined according to World Health Organization criteria (acute onset of neurological deficit persisting for >24 hours in case of a stroke or for <24 hours in case of a TIA),^22^ with review of all cases as soon as possible after presentation by the same senior neurologist (P.M.R.) throughout the study.

All patients received intensive medical management, including dual antiplatelet therapy (aspirin and clopidogrel) for the first month with aspirin or clopidogrel monotherapy thereafter (for lacunar and nonlacunar stroke) and anticoagulation for patients with atrial fibrillation, high-dose statin, and treatment of hypertension to guideline targets (<130/80 mm Hg). Patients were also provided smoking cessation and dietary advice.

Patients were followed up face to face at 1, 6, 12, 24, 60, and 120 months by a study nurse or physician to identify any recurrent stroke (supplemented by a review of primary care records) and to ensure medication compliance and adequate blood pressure control. All recurrent events that occurred during follow-up would also be identified by the ongoing daily case ascertainment. Patients who had moved out of the study area were followed up via telephone at the same points as face-to-face follow-up. We recorded all deaths during follow-up with the underlying causes by direct follow-up, via primary care records, and by centralized registration with the UK Office for National Statistics.

### Imaging Procedures

The MRI scanners and protocols used in OXVASC have been described elsewhere,^23^ but sequences included diffusion-weighted imaging, time-of-flight (TOF) angiography of the intracranial arteries, and gadolinium contrast-enhanced angiography (CE-MRA) of the intracranial and cervicocranial arteries, including the aortic arch. Patients were scanned at the Acute Vascular Imaging Centre of John Radcliffe Hospital (Oxford, England) using a 3-T Siemens Verio scanner; a neurovascular coil was used (CE-MRA sequence: 15 mL of ProHance [Bracco] followed by 40 mL of sodium chloride; flow rate, 2 mL per second; repetition time, 22 milliseconds; echo time, 3.6 milliseconds; flip angle, 18°; slice thickness, 0.5 mm). The CTA was done with a Toshiba, Aquilion 64, 64-slice scanner and transcranial Doppler with Doppler Box (Compumedics DWL).

Reconstructed TOF and CE-MRA sequences were used to assess intracranial and extracranial stenosis, respectively. Both sequences were used for assessing potential artifact. In the case of CTA use, unreconstructed CTA images were analyzed.

Significant stenoses were defined as 50% or more of the luminal diameter measured using the WASID method^24^ between the narrowest point and compared with the normal luminal size before the stenosis, or using Stroke Outcomes and Neuroimaging of Intracranial Atherosclerosis criteria with transcranial Doppler ultrasonography (TCD).^25^ Trained assessors (R.H. and F.J.W.) independently evaluated the images for vascular stenosis and were masked to the clinical details and consultant neuroradiologist report (W.K.). In situations of disagreement, a third assessor adjudicated (L.L.). Arteries assessed included the extracranial (subclavian, common carotid, proximal internal carotid, and vertebral [V1, V2, and V3]) and intracranial (distal internal carotid, middle cerebral [M1 and M2], anterior cerebral, posterior cerebral [P1 and P2], basilar, posterior communicating, and vertebral [V4]). eTable 1 in the Supplement outlines the standard anatomical landmarks used. Interobserver agreement for 50% or greater stenosis was assessed using 50 randomly selected patients who had received MRA or CTA.

All 50% or greater stenoses were classified as symptomatic or asymptomatic in association with the most recent clinical presentation and results of parenchymal brain imaging. Asymptomatic ICS was further classified to only (indicating isolated asymptomatic ICS) or any asymptomatic ICS (indicating coexistent symptomatic and asymptomatic ICS).

### Statistical Analysis

Analyses included all eligible patients with intracranial vascular imaging. Interobserver agreement for 50% or greater stenosis was assessed using the Cohen κ. There was good agreement for the presence of intracranial, extracranial, and no stenosis (Cohen κ: 0.82, 0.79, and 0.84, respectively; n = 50).

Baseline characteristics were compared between patients with asymptomatic ICS vs no stenosis using χ^2^or *t* tests as appropriate. We calculated the age-specific prevalence of asymptomatic ICS in 10-year bands and compared rates with those for asymptomatic extracranial carotid stenosis. We also determined any other predictors of any 50% or greater asymptomatic ICS with univariate and age-adjusted regression analyses.

We used a Kaplan-Meier survival analysis to determine the risk of recurrent ischemic stroke during follow-up after the index event for patients with asymptomatic ICS vs those with no ICS. Analyses were censored at death or the end of follow-up (September 28, 2018). All patients had at least 6 months of follow-up. Patients who also had a recently symptomatic ICS were excluded from our primary analysis of overall stroke risk but were included in the analysis of the risk of stroke in the territory of the asymptomatic ICS (ie, distal to the stenosis).

We used a Cox regression analysis to compare risks of recurrent ischemic stroke, ischemic vascular events (ischemic stroke, myocardial infarction, or peripheral vascular disease), and death during follow-up in patients with asymptomatic ICS vs no ICS with adjustment for baseline characteristics that were independent predictors of the presence of ICS. All statistical analyses were performed with SPSS, version 25.0 (IBM) and statistical significance was set at *P *< .05. Requests for access to the data reported in this article will be considered by the corresponding author.

## Results

Of 1579 eligible patients (1000 [63.3%] with TIA; 579 [36.7%] with minor stroke), 1368 (86.6%) underwent intracranial vascular imaging (1034 [65.5%] via MRA; 253 [16.0%] via CTA; 81 [5.1%] via TCD only), whereas 154 (9.8%) only received carotid bifurcation ultrasonography imaging (often because of contraindications to MRA and CTA) and 57 (3.6%) did not undergo any vascular imaging (eTable 2 in the Supplement). Patients who did not receive intracranial vascular imaging were older with a higher burden of vascular risk factors (eTable 3 in the Supplement). There were differences in the detection rates between MRA and CTA (eTable 4 in the Supplement).

Of the 1368 patients with intracranial vascular imaging results, 426 ICS were identified in 260 patients (19.0%). Of 260 patients with any symptomatic or asymptomatic ICS, 205 (78.9%) received MRA, 51 (19.6%) CTA, and 4 (1.5%) TCD. Of these 260 patients, 58 (4.2%) and 155 (11.3%) had only symptomatic and asymptomatic ICS, respectively, whereas 47 (3.4%) had coexistent symptomatic and asymptomatic ICS. There were 285 asymptomatic ICS in the 202 patients with any asymptomatic ICS (14.8%): 70 (24.6%) in the posterior cerebral, 68 (23.9%) in the middle cerebral, 59 (20.7%) in the intracranial internal carotid, 54 (18.9%) in the intracranial vertebral, and 34 (11.9%) in other intracranial arteries.

Patients with only asymptomatic ICS were older than those without ICS and had a higher burden of hypertension, hyperlipidemia, previous stroke/TIA, peripheral vascular disease, and ischemic heart disease (Table 1). The same trend of associations was seen in those with any asymptomatic ICS (eTable 5 in the Supplement).

**Table 1.** Baseline Characteristics of Patients With Intracranial Vascular Imaging Stratified According to the Presence of 50% or Greater Asymptomatic Intracranial Stenosis

Characteristic	No. (%)		*P* value
	Asymptomatic ICS only (n = 155)^a^	No ICS (n = 1108)^b^	
Age, mean (SD), y	77.3 (10.5)	67.7 (13.8)	<.001
Men	75 (48.4)	558 (50.4)	.65
White	148 (95.5)	1045 (94.3)	.71
Hypertension	110 (71.0)	572 (51.6)	<.001
Diabetes	25 (16.1)	134 (12.1)	.16
Hyperlipidemia	65 (41.9)	355 (32.0)	.01
Current smoker	15 (9.7)	165 (14.9)	.08
Atrial fibrillation	35 (22.6)	145 (13.1)	.002
Any vascular disease^c^	67 (43.2)	259 (23.4)	<.001
History of stroke or TIA	36 (23.2)	143 (12.9)	.001
Peripheral vascular disease	12 (7.7)	36 (3.2)	.01
Ischemic heart disease	34 (21.9)	120 (10.8)	<.001
Event type			
TIA	105 (67.7)	735 (66.3)	.73
Minor stroke	50 (32.3)	373 (33.7)	
TOAST classification			
Cardioembolic	38 (24.5)	172 (15.5)	<.001
Atherosclerotic	19 (12.3)	78 (7.0)	
Undetermined	49 (31.6)	494 (44.6)	
Lacunar	13 (8.4)	120 (10.8)	
Multiple/unknown/other	36 (23.2)	244 (22.1)	
Vascular territory			.92
Carotid	84 (54.2)	580 (51.9)	.92
Vertebrobasilar	53 (34.2)	408 (36.4)	
Uncertain/both	18 (11.6)	120 (11.7)	

Abbreviations: ICS, intracranial stenosis; TIA, transient ischemic attack; TOAST, Trial of Org 10172 in Acute Stroke Treatment.

^a^ Excluding patients with any symptomatic ICS.

^b^ Including patients with less than 50% ICS.

^c^ Vascular disease: ischemic stroke/TIA, peripheral vascular disease, or ischemic heart disease.

**Figure 1.**
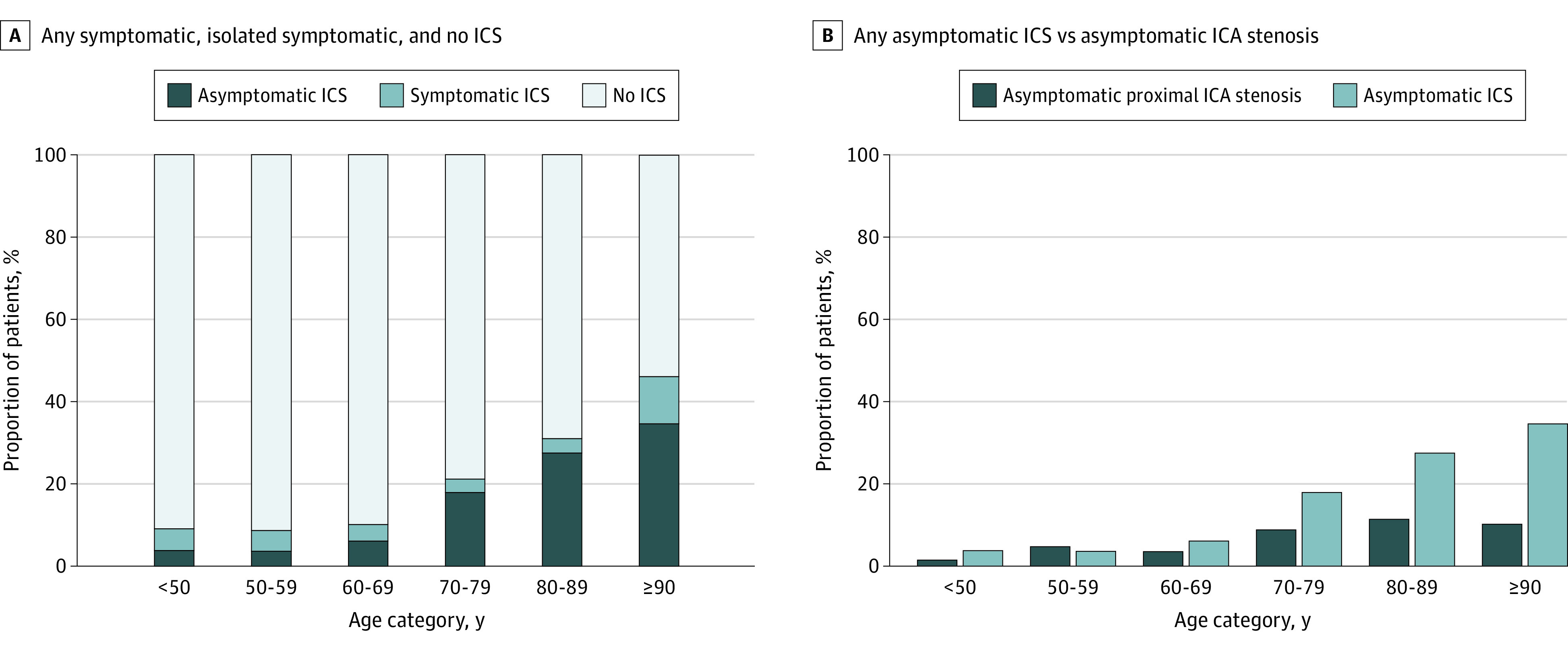
Age-Specific Prevalence of Any Asymptomatic, Isolated Symptomatic, and No Intracranial Stenosis (ICS) and Any Asymptomatic ICS vs Any Asymptomatic Extracranial Internal Carotid Artery (ICA) Stenosis

The prevalence of any asymptomatic ICS increased with age (Figure 1A) from 3.8% for those younger than 50 years to 34.6% for those 90 years or older (*P* for trend < .001; odds ratio per decade, 1.96; 95% CI, 1.69-2.27). The prevalence of any asymptomatic 50% or greater extracranial internal carotid artery stenosis was less than that of any asymptomatic ICS in all age categories (Figure 1B), from 1.5% for those younger than 50 years to 10.2% for those 90 years or older (202 [14.8%] vs 105 [7.2%]; relative risk, 2.04; 95% CI, 1.63-2.55; *P* < .001). Only older age (odds ratio, 1.80; 95% CI, 1.51-2.14) independently predicted only asymptomatic ICS (Table 2), whereas older age, hypertension, and prior stroke/TIA were independent predictors of any asymptomatic ICS (eTable 6 in the Supplement).

**Table 2.** Differences in Characteristics of 155 Patients With Only Asymptomatic Intracranial Stenosis vs 1108 Patients With No Intracranial Stenosis on Univariate and Adjusted Regression Analyses

Characteristic	Unadjusted OR (95% CI)	*P* value	Age-adjusted OR (95% CI)	*P* value	Multivariable-adjusted OR (95% CI)^a^	*P* value
Age (per 10 y)	1.95 (1.66-2.29)	<.001	NA	NA	1.80 (1.51- 2.14)	<.001
Men	0.92 (0.66-1.29)	.65	1.11 (0.78-1.57)	.56	1.02 (0.71-1.47)	.91
Hypertension	2.29 (1.59-3.30)	<.001	1.57 (1.07-2.30)	.02	1.39 (0.93-2.08)	.11
Diabetes	1.40 (0.88-2.22)	.16	1.32 (0.82-2.13)	.26	1.15 (0.70-1.91)	.58
Hyperlipidemia	1.53 (1.09-2.16)	.02	1.33 (0.93-1.89)	.12	1.06 (0.72-1.56)	.78
Atrial fibrillation	1.94 (1.28-2.93)	.002	1.32 (0.86-2.04)	.21	1.24 (0.80-1.93)	.34
Any prior vascular disease^b^	2.50 (1.76-3.53)	<.001	1.67 (1.16- 2.41)	.01	NA	NA
Prior stroke/TIA	2.04 (1.35-3.08)	.001	1.55 (1.01-2.39)	.045	1.45 (0.93-2.27)	.10
Peripheral vascular disease	2.50 (1.27-4.91)	.01	1.72 (0.85-3.45)	.13	1.44 (0.70-2.97)	.32
Ischemic heart disease	2.31 (1.51-3.54)	<.001	1.54 (0.99-2.40)	.06	1.36 (0.85-2.17)	.20
Event type						
TIA	1 [Reference]	NA	1 [Reference]	NA	1 [Reference]	NA
Minor stroke	0.94 (0.66-1.34)	.73	0.99 (0.68-1.43)	.95	0.97 (0.66-1.42)	.87

Abbreviations: NA, not applicable; OR, odds ratio; TIA, transient ischemic attack.

^a^ Adjusted for all variables in the table except any prior vascular disease.

^b^ Vascular disease: ischemic stroke/TIA, peripheral vascular disease, or ischemic heart disease.

Of 155 of 1368 patients with only asymptomatic ICS (11.3%), none were lost to follow-up and 8 of 155 (5.2%) had recurrent ischemic strokes during 506 patient-years of follow-up; the 7-year risk of recurrent ischemic stroke was 6.8% (95% CI, 1.7-11.9). Only 3 of these strokes were in the territory of the previously asymptomatic ICS (annualized risk, 0.59%; 95% CI, 0.12-1.73). In the same period, 28 of 155 patients (18.1%) died and 11 (7.1%) had recurrent ischemic events (including 2 myocardial infarctions and one peripheral vascular event). During follow-up, there was also no difference in risk of recurrent ischemic stroke, death, or any vascular event (ischemic stroke, peripheral vascular disease, or ischemic heart disease) for the 155 patients with only asymptomatic ICS (Table 3).

**Table 3.** Risk of Outcomes During Follow-up in 155 Patients With Only Asymptomatic Intracranial Stenosis vs Patients With No Intracranial Stenosis

Outcome	ICS		No ICS		Unadjusted HR (95% CI)	*P* value	Age-adjusted HR (95% CI)	*P* value
	Event	Total	Event	Total				
Ischemic stroke	8	155	57	1108	1.03 (0.49-2.17)	.93	0.80 (0.38-1.71)	.57
Death	28	155	115	1108	1.85 (1.22-2.79)	.004	1.06 (0.69-1.60)	.80
Any ischemic event^a^	11	155	76	1108	1.04 (0.55-1.96)	.90	0.78 (0.41-1.49)	.45

Abbreviations: HR, hazard ratio; ICS, intracranial stenosis.

^a^ Any of ischemic stroke, peripheral vascular disease, or ischemic heart disease.

**Figure 2.**
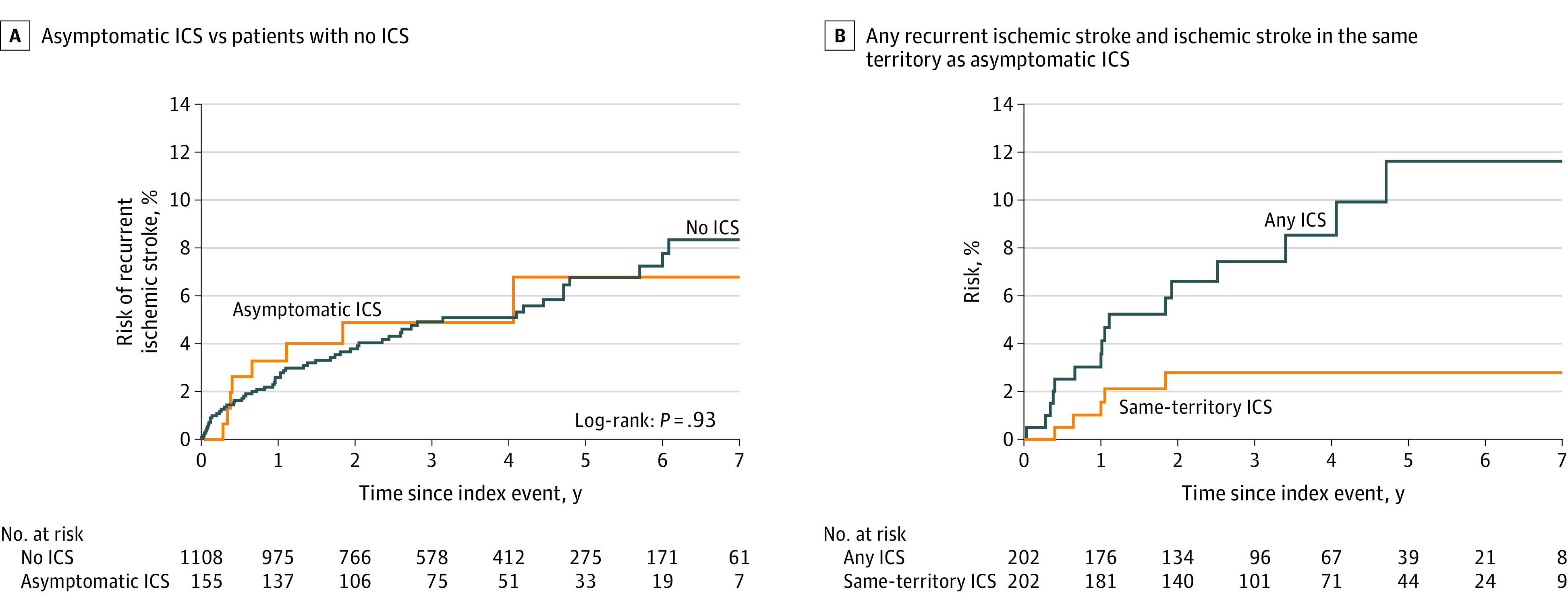
Seven-Year Risk of Recurrent Ischemic Stroke in Patients With Only Asymptomatic Intracranial Stenosis (ICS) vs Patients With No ICS and 7-Year Risk of Any Recurrent Ischemic Stroke and Ischemic Stroke in the Same Territory as the Asymptomatic ICS in 202 Patients With Any Asymptomatic ICS

There was no difference in the risk of recurrent ischemic stroke between patients with only asymptomatic ICS and no ICS (Figure 2A) overall or after excluding patients with atrial fibrillation (eFigure in the Supplement). Figure 2B shows the risk of recurrent stroke during follow-up in the 202 patients with any asymptomatic ICS (14.8%) for risk of any ischemic stroke in any vascular territory (n = 16 events) and for risk of recurrent ischemic stroke distal to the asymptomatic ICS (n = 5 events).

## Discussion

In this population-based study of predominantly white patients with ischemic stroke/TIA and a high rate of intracranial imaging, we found a prevalence of 14.8% for any asymptomatic ICS, with the highest rates at older ages. The prevalence of 50% or greater asymptomatic ICS was approximately double that of 50% or greater asymptomatic extracranial internal carotid artery stenosis. However, the prognosis of asymptomatic ICS was relatively benign while receiving medical treatment alone, with no increase in risk of recurrent ischemic stroke, major ischemic vascular events, or death compared with patients with no ICS. The annual risk of any recurrent ischemic stroke in patients with asymptomatic ICS was about 1%, falling to about 0.6% per year for ischemic stroke in the same territory of the previously asymptomatic ICS.

Some patients had symptomatic and asymptomatic ICS. We excluded these patients from our main analysis of risk of all recurrent ischemic stroke because symptomatic ICS conveys an increased risk of recurrent ischemic stroke.^26,27^ However, our sensitivity analysis of the prognosis of any asymptomatic ICS (Figure 2B), which included patients with symptomatic and asymptomatic ICS, confirmed a low risk of ischemic stroke in the same territory of the previously asymptomatic ICS.

The clinical implication of our findings is that no specific additional treatments or follow-up imaging are routinely required for patients with TIA/stroke with asymptomatic ICS. Patients with these imaging findings should be treated per standard guidelines for stroke secondary prevention.^28,29^ Furthermore, patients can be advised that asymptomatic ICS does not convey an increased risk of recurrent ischemic stroke, vascular event, or death beyond those patients with minor stroke/TIA without ICS.

### Strengths and Limitations

The strengths of our study include its large, population-based nature with high rates of ascertainment, complete follow-up, thorough investigation, and intensive medical management. Nearly 90% of eligible patients underwent intracranial vascular imaging, with most receiving MRA. We chose the most commonly used definition of ICS (≥50% luminal stenosis) and demonstrated good interrater reliability.

However, our study also had some limitations. First, our findings do not apply to patients of color who have higher rates of ICS or patients with major stroke. However, 90% of all recurrent strokes occur in patients with a previous TIA/ minor stroke as opposed to a major stroke.^30^ Similarly, although we stratified our analysis by the presence of atrial fibrillation, we were not powered to examine the risks of asymptomatic ICS in other etiological stroke types. Second, although the patients were followed up regularly by study nurses and clinicians offering similar lifestyle advice and risk factor management to that of previous trials, the rates of medication compliance and risk factor control are likely to have been higher than in some other settings. Third, although two-thirds of patients in our study received our first preference of MRI/MRA brain imaging, other imaging modalities had to be used when MRA was contraindicated, principally CTA, which has a different sensitivity and specificity for detecting ICS. Although we did find differences in the detection rates between MRA and CTA (eTable 4 in the Supplement), CTA was used for an older subgroup of patients with contraindications to MRI (such as pacemakers) and with a higher burden of vascular risk factors. However, many previous studies have relied on only TCD screening, which has a much lower sensitivity.^9,31^ Fourth, 13.4% of patients in the cohort did not receive intracranial vascular imaging; these patients were older and had a higher burden of vascular risk factors. This may underestimate the prevalence and prognosis of asymptomatic ICS, especially at older ages. Fifth, TOF MRA is prone to artifact because of flow abnormalities; low flow may mimic stenosis and high flow through stenosis may underestimate its degree.^32^ However, we used a combination of contrast-enhanced and TOF MRA to improve the specificity of ICS detection, and MRA is commonly used clinically to detect ICS. Catheter angiography has a greater sensitivity and would permit further exploration of ICS grades, but the associated risks preclude its use for screening. Finally, the mean follow-up in our study of 3 years might underestimate longer-term risk and further studies will be required to evaluate this.

## Conclusions

Asymptomatic ICS is twice as prevalent in white patients with minor stroke/TIA as asymptomatic carotid stenosis, but the risk of recurrent ischemic stroke appears to be no higher than in patients without ICS. These results should reassure clinicians and patients that no additional management strategy or follow-up is required routinely for this commonly identified pathology.

## Notes

Supplement. **eTable 1. **Standard anatomical landmarks used to locate extra- and intracranial stenoses. **eTable 2. **Details of imaging received by study patients and reasons for non-imaging. **eTable 3. **Baseline characteristics of study population, stratified by intracranial vascular imaging. **eTable 4. **Distribution of any intracranial stenosis (ICS) and extracranial vascular stenosis stratified by magnetic resonance angiography (MRA) and computed tomography angiography (CTA). **eTable 5**. Baseline characteristics of the study population stratified according to the presence of any asymptomatic intracranial stenosis (ICS). **eTable 6***. *Predictors of any asymptomatic intracranial stenosis in the study population. **eFigure. **The 7-year risk of recurrent ischemic stroke in patients with only asymptomatic intracranial stenosis (ICS) versus patients with no ICS, excluding those with atrial fibrillation.
