# Supplementary material for: Prognosis of Asymptomatic Intracranial Stenosis in Patients With Transient Ischemic Attack and Minor Stroke
Source: JAMA Neurol. 2020 May 26;77(8):1–8. doi: 10.1001/jamaneurol.2020.1326 (PMC7251503; doi:10.1001/jamaneurol.2020.1326)
Supplement: Supplement. — eTable 1. Standard anatomical landmarks used to locate extra- and intracranial stenoses. eTable 2. Details of imaging received by study patients and reasons for non-imaging. eTable 3. Baseline characteristics of study population, stratified by intracranial vascular imaging. eTable 4. Distribution of any intracranial stenosis (ICS) and extracranial vascular stenosis stratified by magnetic resonance angiography (MRA) and computed tomography angiography (CTA). eTable 5. Baseline characteristics of the study population stratified according to the presence of any asymptomatic intracranial stenosis (ICS). eTable 6. Predictors of any asymptomatic intracranial stenosis in the study population. eFigure. The 7-year risk of recurrent ischemic stroke in patients with only asymptomatic intracranial stenosis (ICS) versus patients with no ICS, excluding those with atrial fibrillation. [file jamaneurol-77-947-s001.pdf]

Supplementary Online Content

Hurford R, Wolters FJ, Li L, Lau KK, Küker W, Rothwell PM. Prognosis of asymptomatic intracranial stenosis in patients with transient ischemic attack and minor stroke. *JAMA Neurol*. Published online May 26, 2020. doi:10.1001/jamaneurol/2020.1326

eTable 1. Standard anatomical landmarks used to locate extra- and intracranial stenoses.

eTable 2. Details of imaging received by study patients and reasons for non-imaging.

eTable 3. Baseline characteristics of study population, stratified by intracranial vascular imaging.

eTable 4. Distribution of any intracranial stenosis (ICS) and extracranial vascular stenosis stratified by magnetic resonance angiography (MRA) and computed tomography angiography (CTA).

eTable 5. Baseline characteristics of the study population stratified according to the presence of any asymptomatic intracranial stenosis (ICS).

eTable 6. Predictors of any asymptomatic intracranial stenosis in the study population.

eFigure. The 7-year risk of recurrent ischemic stroke in patients with only asymptomatic intracranial stenosis (ICS) versus patients with no ICS, excluding those with atrial fibrillation.

This supplementary material has been provided by the authors to give readers additional information about their work.

© Hurford R et al. *JAMA Neurology*.

eTable 1: Standard anatomical landmarks used to locate extra- and intracranial stenoses.

Vessel	Anatomical landmark
Subclavian artery	Right – from the brachiocephalic trunk (left - from the aortic arch) to the lateral border of the first rib.
Common carotid artery	Right – from the brachiocephalic trunk (left - from the aortic arch) to the terminal internal and external carotid artery branches.
Cervical internal carotid artery (ICA)	Left and right. Cervical segment – terminal branch of common carotid artery to petrous portion of temporal bone.
Distal internal carotid artery (ICA)	Left and right. C2 to C7 – petrous portion of temporal bone to the terminal branching of ACA and MCA.
Middle cerebral artery (MCA) M1	Left and right terminal bifurcation of ICA until bifurcation to superior or inferior divisions.
Middle cerebral artery (MCA) M2	From the main bifurcation of MCA coursing posterosuperiorly within the insula cleft to circular sulcus of insula.
Anterior cerebral artery (ACA)	Left and right branches of ICA. A1, A2 and anterior communicating subdivisions considered as one.
Posterior communicating artery (Pcomm)	Left and right from the distal ICA (prior to the ACA and MCA bifurcation) to the PCA P1 segment.
Posterior cerebral artery (PCA) P1	Left and right terminal branches of the basilar artery to the Pcomm in interpeduncular cistern.
Posterior cerebral artery (PCA) P2	Left and right. From P1 Pcomm junction to quadrigeminal cistern.
Basilar artery (BA)	Confluence of left and right V4 to terminal bifurcation of left and right PCA.
Vertebral artery (VA) V1	Left and right – origin from subclavian arteries to transverse foramen of C6 vertebrae.
Vertebral artery (VA) V2	Left and right – from transverse foramen of C6 vertebrae to transverse foramen of C2.
Vertebral artery (VA) V3	Left and right – from transverse foramen of C2 to dura.
Vertebral artery (VA) V4	Left and right – from dura to basilar artery formation (or termination as PICA).

© Hurford R et al. *JAMA Neurology*.

eTable 2: Details of imaging received by study patients and reasons for non-imaging.

**Imaging modality**	**Patients (%)**<br>**N= 1579**
**Intracranial vascular imaging**	
Magnetic resonance angiography	1034 (65.5)
Computed tomography angiography	253 (16.0)
Transcranial Doppler ultrasound	81 (5.2)
**Extracranial vascular imaging only**	
Carotid Doppler ultrasound	154 (9.8)
**No vascular imaging**	57 (3.6)
Known atrial fibrillation	25
Previously investigated or imaged elsewhere	8
Other clinical or logistical issue	24

eTable 3: Baseline characteristics of study population, stratified by intracranial vascular imaging.

	Intracranial vascular imaging (N= 1368)	***No intracranial vascular imaging (N= 211)***	**p-value**
Mean age (SD)	69.2 (13.9)	76.5 (12.1)	<0.0001
Male sex (%)	700 (51.2)	110 (52.1)	0.82
Caucasian (%)	1291 (94.4)	201 (95.3)	0.75
Hypertension (%)	754 (55.1)	147 (69.7)	<0.0001
Diabetes mellitus (%)	183 (13.4)	48 (22.7)	0.0004
Hyperlipidaemia (%)	466 (34.1)	79 (37.4)	0.35
Current smoker (%)	193 (14.1)	25 (11.8)	0.45
Atrial fibrillation (%)	204 (14.9)	57 (27.0)	<0.0001
Any vascular disease[§] (%)	373 (27.3)	106 (50.2)	<0.0001
History of stroke or TIA (%)	204 (14.9)	76 (36.0)	<0.0001
PVD (%)	61 (4.5)	17 (8.1)	0.038
IHD (%)	179 (13.1)	52 (24.6)	0.0005
Event type			
TIA (%)	892 (65.2)	108 (51.2)	<0.0001
Minor stroke (%)	476 (34.8)	103 (48.8)	
TOAST classification			
Cardioembolic (%)	210 (15.4)	69 (32.7)	0.15
Atherosclerotic (%)	181 (13.2)	21 (10.0)	
Undetermined (%)	543 (39.7)	35 (16.6)	
Lacunar (%)	133 (9.7)	24 (11.4)	
Multiple/unknown/other (%)	301 (22.0)	62 (29.3)	
Vascular territory			
Carotid (%)	716 (52.3)	148 (70.1)	<0.0001
Vertebrobasilar (%)	504 (36.8)	37 (17.5)	
Uncertain/ both (%)	148 (10.8)	26 (12.3)	

PVD= peripheral vascular disease, IHD= ischaemic heart disease. [§]vascular disease = ischaemic stroke/ TIA, PVD or IHD.

© Hurford R et al. *JAMA Neurology*.

eTable 4: Distribution of any asymptomatic or symptomatic ≥50% intracranial stenosis (ICS) and extracranial vascular stenosis stratified by magnetic resonance angiography (MRA) and computed tomography angiography (CTA).

	**Affected vessels / total number of vessels imaged (%)**[¶]	**MRA, 1304 patients**	**CTA, 253 patients**	**p-value**
**Intracranial vessels**				
Distal ICA	88/ 2458 (3.6)	52/ 1954 (2.7)	36/ 504 (7.1)	<0.0001
ACA	31/ 2565 (1.1)	30/ 2059 (1.5)	1/ 506 (0.2)	0.031
MCA (M1+M2)	104/ 2573 (4.0)*	89/ 2063 (4.3)	11/ 506 (2.2)	0.048
PCA (P1+P2)	98/ 2559 (3.8)	87/ 2053 (4.2)	11/ 506 (2.2)	0.024
PComm	3/ 847 (0.4)	3/ 685 (0.4)	0/ 162 (0)	0.99
Basilar	13/ 1285 (1.0)	11/ 1032 (1.1)	2/ 253 (0.8)	0.99
Vertebral (V4)	98/ 2557 (3.8)	74/ 2051 (3.6)	24/ 506 (4.7)	0.072
**Extracranial vessels**				
Subclavian	76/ 2424 (3.1)	69/ 1944 (3.6)	7/ 480 (1.5)	0.018
Common carotid	34/ 2900 (1.2)[¤]	11/ 1960 (0.6)	22/ 491 (4.5)	<0.0001
Proximal ICA	273/ 2867 (9.5)[¥]	132/1960(6.7)	67/491 (13.6)	<0.0001
Vertebral (V1-3)	236/ 2447 (9.6)	200/1949 (10.3)	36/469 (7.7)	0.10

*ICA= internal carotid artery; ACA = anterior cerebral artery; MCA = medial cerebral artery; PCA = posterior cerebral artery; PComm = posterior communicating artery. ¶A single ICS affecting two contiguous vessel segments are counted as two. *Including 4 MCA ICS detected by transcranial Doppler. ¤ Including carotid Doppler ultrasound 1 / 449; ¥including carotid Doppler ultrasound 74 / 416.*

eTable 5: Baseline characteristics of the study population stratified according to the presence of any asymptomatic intracranial stenosis (ICS).

**Characteristic**	**Patients with intracranial vascular imaging and any asymptomatic ICS* (N= 1,308)**		**p-value**
	Any asymptomatic ICS (N= 202)	*No ICS (N= 1,108)*	
Mean age (SD)	77.3 (10.5)	67.7 (13.8)	<0.0001
Male sex (%)	107 (53.0)	558 (50.4)	0.50
Caucasian (%)	193 (95.5)	1045 (94.3)	0.93
Hypertension (%)	149 (73.8)	572 (51.6)	<0.0001
Diabetes mellitus (%)	39 (19.3)	134 (12.1)	0.005
Hyperlipidaemia (%)	90 (44.6)	355 (32.0)	0.001
Current smoker (%)	20 (9.9)	165 (14.9)	0.060
Atrial fibrillation (%)	47 (23.3)	145 (13.1)	<0.0001
Any vascular disease§ (%)	93 (46.0)	259 (23.4)	<0.0001
History of stroke or TIA (%)	49 (24.3)	143 (12.9)	<0.0001
PVD (%)	19 (9.4)	36 (3.2)	<0.0001
IHD (%)	50 (24.8)	120 (10.8)	<0.0001
Event type			
TIA (%)	127 (62.9)	735 (66.3)	0.34
Minor stroke (%)	75 (37.1)	373 (33.7)	
TOAST classification			
Cardioembolic (%)	38 (18.8)	172 (15.5)	<0.0001
Atherosclerotic (%)	56 (27.7)	78 (7.0)	
Undetermined (%)	49 (24.3)	494 (44.6)	
Lacunar (%)	13 (6.4)	120 (10.8)	
Multiple/unknown/other (%)	46 (22.8)	244 (22.1)	
Vascular territory			
Carotid (%)	110 (54.5)	580 (51.9)	0.89
Vertebrobasilar (%)	74 (36.6)	408 (36.4)	
Uncertain/ both (%)	18 (8.9)	120 (11.7)	

**Including: MRA, CTA, TCD. PVD= peripheral vascular disease, IHD= ischaemic heart disease. §vascular disease = ischaemic stroke/ TIA, PVD or IHD.*

© Hurford R et al. *JAMA Neurology*.

eTable 6: Predictors of any asymptomatic intracranial stenosis in the study population.

	Unadjusted risk indicators OR (95% CI)	p-value	Age-adjusted risk indicators OR (95% CI)	p-value	Multivariable risk indicators OR (95% CI)	p-value
Age (per 10 years)	1.96 (1.69-1.27)	<0.0001	-		**1.80 (1.54-2.11)**	**<0.0001**
Male sex	1.11 (0.82-1.50)	0.50	1.36 (0.99-1.87)	0.056	1.21 (0.87-1.68)	0.26
Hypertension	2.63 (1.89-3.68)	<0.0001	1.84 (1.30-2.61)	0.001	**1.55 (1.07-2.25)**	**0.019**
Diabetes mellitus	1.74 (1.17-2.58)	0.006	1.67 (1.11-2.52)	0.015	1.32 (0.85-2.04)	0.22
Hyperlipidaemia	1.70 (1.26-2.31)	0.001	1.48 (1.08-2.03)	0.015	1.05 (0.74-1.50)	0.77
Atrial fibrillation	2.01 (1.39-2.92)	<0.0001	1.34 (0.91-1.98)	0.14	1.20 (0.81-1.79)	0.37
Any prior vascular disease*	2.80 (2.05-3.81)	<0.0001	1.90 (1.37-2.63)	<0.0001	-	
Prior stroke/ TIA	2.16 (1.50-3.12)	<0.0001	1.68 (1.15-2.47)	0.008	**1.58 (1.05-2.34)**	**0.027**
PVD	3.09 (1.74-5.51)	<0.0001	2.10 (1.15-3.83)	0.016	1.60 (0.86-2.98)	0.14
IHD	2.71 (1.87-3.93)	<0.0001	1.81 (1.23-2.68)	0.003	1.44 (0.95-2.18)	0.087
Event type						
TIA	REFERENCE		REFERENCE		REFERENCE	
Minor stroke	1.16 (0.85-1.59)	0.34	1.23 (0.89-1.70)	0.21	1.19 (0.85-2.66)	0.031

*PVD= peripheral vascular disease; IHD= ischaemic heart disease. *vascular disease = ischaemic stroke/ TIA, PVD or IHD.*

© Hurford R et al. *JAMA Neurology*.

eFigure 1: The 7-year risk of recurrent ischemic stroke in patients with only asymptomatic intracranial stenosis (ICS) versus patients with no ICS, excluding those with atrial fibrillation

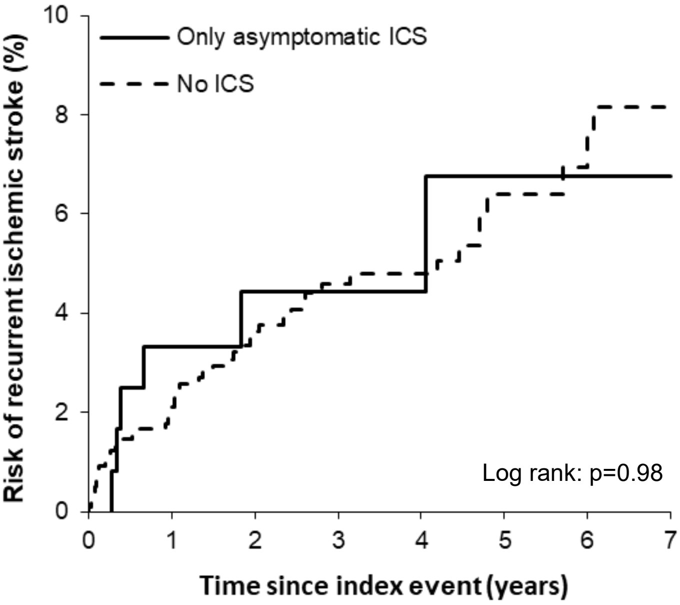

No ICS	963	858	676	512	362	246	155	56
Asymptomatic ICS	120	109	85	56	40	24	14	5

© Hurford R et al. *JAMA Neurology*.

Supplementary References

1. Wityk RJ, Lehman D, Klag M, Coresh J, Ahn H, Litt B. Race and sex differences in the distribution of cerebral atherosclerosis. *Stroke*. 1996;27(11):1974-1980.

2. Thijs VN, Albers GW. Symptomatic intracranial atherosclerosis: outcome of patients who fail antithrombotic therapy. *Neurology*. 2000;55(4):490-497. doi:10.1212/wnl.55.4.490

3. Nahab F, Cotsonis G, Lynn M, et al. Prevalence and prognosis of coexistent asymptomatic intracranial stenosis. *Stroke*. 2008;39(3):1039-1041. doi:10.1161/STROKEAHA.107.499475

4. Meseguer E, Lavallee PC, Mazighi M, et al. Yield of systematic transcranial Doppler in patients with transient ischemic attack. *Ann Neurol*. 2010;68(1):9-17. doi:10.1002/ana.21921

5. Lau AY, Wong KL, Lev M, Furie K, Smith W, Kim AS. Burden of intracranial steno-occlusive lesions on initial computed tomography angiography predicts poor outcome in patients with acute stroke. *Stroke*. 2013;44(5):1310-1316. doi:10.1161/STROKEAHA.111.672741

6. Homburg PJ, Plas GJJ, Rozie S, van der Lugt A, Dippel DWJ. Prevalence and calcification of intracranial arterial stenotic lesions as assessed with multidetector computed tomography angiography. *Stroke*. 2011;42(5):1244-1250. doi:10.1161/STROKEAHA.110.596254

7. von Weitzel-Mudersbach P, Paaske Johnsen S, Andersen G. Intra- and extracranial stenoses in TIA - findings from the Aarhus TIA-study: a prospective population-based study. *Persecptives Med*. 2012;1:207-210.

8. Ovesen C, Abild A, Christensen AF, et al. Prevalence and long-term clinical significance of intracranial atherosclerosis after ischaemic stroke or transient ischaemic attack: a cohort study. *BMJ Open*. 2013;3(10):e003724. doi:10.1136/bmjopen-2013-003724

9. Logallo N, Naess H, Waje-Andreassen U, Thomassen L. Prevalence of intracranial stenosis in a Norwegian ischemic stroke population. *J Stroke Cerebrovasc Dis*. 2014;23(6):1611-1615. doi:10.1016/j.jstrokecerebrovasdis.2013.12.049

10. Tsivgoulis G, Vadikolias K, Heliopoulos I, et al. Prevalence of symptomatic intracranial atherosclerosis in Caucasians: a prospective, multicenter, transcranial Doppler study. *J Neuroimaging*. 2014;24(1):11-17. doi:10.1111/j.1552-6569.2012.00707.x

11. Gouveia A, Sargento-Freitas J, Penetra J, et al. Recurrence in intracranial atherosclerotic disease: a stenosis-based analysis. *J Stroke Cerebrovasc Dis*. 2014;23(8):2080-2084. doi:10.1016/j.jstrokecerebrovasdis.2014.03.015

12. Hoshino T, Sissani L, Labreuche J, et al. Prevalence of Systemic Atherosclerosis Burdens and Overlapping Stroke Etiologies and Their Associations With Long-term Vascular Prognosis in Stroke With Intracranial Atherosclerotic Disease. *JAMA Neurol*. 2018;75(2):203-211. doi:10.1001/jamaneurol.2017.3960

13. Kappelle LJ, Eliasziw M, Fox AJ, Sharpe BL, Barnett HJ. Importance of intracranial atherosclerotic disease in patients with symptomatic stenosis of the internal carotid artery. The North American Symptomatic Carotid Endarterectomy Trail. *Stroke*. 1999;30(2):282-286. doi:10.1161/01.str.30.2.282

14. von Sarnowski B, Schminke U, Tatlisumak T, et al. Prevalence of stenoses and occlusions of brain-supplying arteries in young stroke patients. *Neurology*. 2013;80(14):1287-1294. doi:10.1212/WNL.0b013e31828ab2ed

15. Mattioni A, Cenciarelli S, Biessels G, van Seeters T, Algra A, Ricci S. Prevalence of intracranial large artery stenosis and occlusion in patients with acute ischaemic stroke or TIA. *Neurol Sci Off J Ital Neurol Soc Ital Soc Clin Neurophysiol*. 2014;35(3):349-355. doi:10.1007/s10072-013-1516-4

16. Kremer C, Schaettin T, Georgiadis D, Baumgartner RW. Prognosis of asymptomatic stenosis of the middle cerebral artery. *J Neurol Neurosurg Psychiatry*. 2004;75(9):1300-1303. doi:10.1136/jnnp.2003.017863

© Hurford R et al. *JAMA Neurology*.

Oxford Vascular Study group members (current research staff)

Name	Role	Institution
Prof Peter M Rothwell	Director	University of Oxford
Dr Louise Silver	Research Coordinator	University of Oxford
Prof Sarah Pendlebury	Senior Research Fellow	University of Oxford
Dr Wilhelm Kuker	Neuroradiologist	University of Oxford
Dr Alastair Webb	Senior Research Fellow	University of Oxford
Dr Linxin Li	Senior Research Fellow	University of Oxford
Dr Sara Mazzucco	Senior Research Fellow	University of Oxford
Dr Gabriel Yiin	Senior Research Fellow	University of Oxford
Dr Maria Tuna	Senior Research Fellow	University of Oxford
Dr Ramon Luengo-Fernandez	Senior Research Fellow	University of Oxford
Dr Lucy Binney	Senior Research Fellow	University of Oxford
Dr Joanna Moschandreas	Senior Statistician	University of Oxford
Dr Sergei Gutnikov	Database Manager	University of Oxford
Ms Maxine Florey	Research Secretary	University of Oxford
Ms Samantha Carter	Data Manager	University of Oxford
Dr Aubretia McColl	Clinical Research Fellow	University of Oxford
Dr Iain McGurgan	Clinical Research Fellow	University of Oxford
Dr Robert Hurford	Clinical Research Fellow	University of Oxford
Dr Dearbhla Kelly	Clinical Research Fellow	University of Oxford
Dr Cyrus Daruwalla	Clinical Research Fellow	University of Oxford
Ms Sarah Welch	Research Nurse	University of Oxford
Ms Michelle Wilson	Research Physiotherapist	University of Oxford
Ms Fiona Cuthbertson	Research Physiotherapist	University of Oxford
Ms Ellen McCulloch	Research Nurse	University of Oxford
Ms Sally Beebe	Research Nurse	University of Oxford
Ms Karen Bowsher-Brown	Research Nurse	University of Oxford
Ms Josephine Edens	Research Nurse	University of Oxford
Ms Emily-Rose Vaughan-Fowler	Research Nurse	University of Oxford
Ms Anne-Marie Haigh	Research Nurse	University of Oxford
Ms Doina-Amalia Bubulete	Research Nurse	University of Oxford
Ms Amy Lawson	Research Assistant	University of Oxford
Dr Annette Burgess	Laboratory Manager	University of Oxford
Ms Deborah Poole	Laboratory Technician	University of Oxford
Ms Julia Duerden	Laboratory Technician	University of Oxford

© Hurford R et al. *JAMA Neurology*.
